# Phosphorylation of insulin receptor substrates (IRS-1 and IRS-2) is attenuated following cecal ligation and puncture in mice

**DOI:** 10.1186/s10020-023-00703-9

**Published:** 2023-08-07

**Authors:** Deepa Mathew, Julia Barillas-Cerritos, Ana Nedeljkovic-Kurepa, Mabel Abraham, Matthew D. Taylor, Clifford S. Deutschman

**Affiliations:** 1 Department of Pediatrics, Cohen Children’s Medical Center, Lake Success, NY USA; 2 Institute for Molecular Medicine, Feinstein Institutes for Medical Research, Room 3140, 350 Community Dr, Manhasset, NY 11030 USA; 3 Present Address: Pediatric Endocrinology, Metabolism and Diabetes, Winthrop Pediatrics Associates, Mineola, NY USA; 4 Zucker School of Medicine at Hofstra/Northwell, Hempstead, NY USA

**Keywords:** Sepsis, Cecal ligation and puncture, CLP, Insulin, Insulin receptor substrate, Tyrosine phosphorylation, Hypoglycemia, Insulin resistance, Liver, Skeletal muscle

## Abstract

**Background:**

Sepsis is characterized as an insulin resistant state. However, the effects of sepsis on insulin’s signal transduction pathway are unknown. The molecular activity driving insulin signaling is controlled by tyrosine phosphorylation of the insulin receptor β-subunit (IRβ) and of insulin receptor substrate molecules (IRS) -1 and IRS-2.

**Hypothesis:**

Cecal ligation and puncture (CLP) attenuates IRβ, IRS-1 and IRS-2 phosphorylation.

**Methods:**

IACUC-approved studies conformed to ARRIVE guidelines. CLP was performed on C57BL/6 mice; separate cohorts received intraperitoneal insulin at baseline (T_0_) or at 23 or 47 h. post-CLP, 1 h before mice were euthanized. We measured levels of (1) glucose and insulin in serum, (2) IRβ, IRS-1 and IRS-2 in skeletal muscle and liver homogenate and (3) phospho-Irβ (pIRβ) in liver and skeletal muscle, phospho-IRS-1 (pIRS-1) in skeletal muscle and pIRS-2 in liver. Statistical significance was determined using ANOVA with Sidak’s post-hoc correction.

**Results:**

CLP did not affect the concentrations of IRβ, IRS-1or IRS-2 in muscle or liver homogenate or of IRS-1 in liver. Muscle IRS-1 concentration at 48 h. post-CLP was higher than at T_0_. Post-CLP pIRS-1 levels in muscle and pIRβ and pIRS-2 levels in liver were indistinguishable from T_0_ levels. At 48 h. post-CLP pIRβ levels in muscle were higher than at T_0_. Following insulin administration, the relative abundance of pIRβ in muscle and liver at T_0_ and at both post-CLP time points was significantly higher than abundance in untreated controls. In T_0_ controls, the relative abundance of pIRS-1 in muscle and of pIRS-2 in liver following insulin administration was higher than in untreated mice. However, at both post-CLP time points, the relative abundance of pIRS-1 in muscle and of pIRS-2 in liver following insulin administration was not distinguishable from the abundance in untreated mice at the same time point. Serum glucose concentration was significantly lower than T_0_ at 24 h., but not 48 h., post-CLP. Glucose concentration was lower following insulin administration to T_0_ mice but not in post-CLP animals. Serum insulin levels were significantly higher than baseline at both post-CLP time points.

**Conclusions:**

CLP impaired insulin-induced tyrosine phosphorylation of both IRS-1 in muscle and IRS-2 in liver. These findings suggest that the molecular mechanism underlying CLP-induced insulin resistance involves impaired IRS-1/IRS-2 phosphorylation.

## Introduction

Sepsis dysregulates glucose metabolism (Berghe et al. 2001). Indeed, in both septic humans and animal models, the amount of insulin required to reduce blood glucose levels is markedly higher than that needed to achieve the same decrease in the unaffected (Berghe et al. 2001; Clemens et al. 1984). This finding has been termed “insulin resistance”. However, the endocrine milieu of sepsis makes use of this term problematic. In particular, levels of glucagon, epinephrine and cortisol, which enhance glycogen breakdown and gluconeogenesis, are elevated in sepsis (Téblick et al. 2022). Further, sepsis-induced release of several cytokines also affects blood glucose levels (Niekerk and Engelbrecht 2018). Therefore, the high doses of insulin required to correct sepsis-induced hyperglycemia may well reflect the interplay of several different factors. Ultimately, the complex endocrine/inflammatory milieu of sepsis makes it difficult to determine if direct insulin resistance is truly present.

Sepsis also limits endocrine responses (Ingels et al. 2018). This finding in part reflects impaired hormonal activity in cells (Téblick et al. 2022). Serum hormones levels are elevated, perhaps as part of a reflex arc to compensate for the lack of cellular responses. These reduced responses are often associated with a defect in the ability of hormones to affect cellular signal transduction pathways, and in particular with attenuated phosphorylation in these pathways (Yang et al. 1997; Rehman et al. 2020; Yumet et al. 2006; Hsu et al. 1999).

The initial step in the insulin signaling pathway is hormone binding to a dimeric receptor containing α and β subunits (White and Kahn 2021). Insulin binding activates tyrosine kinase activity in the β subunit (IRβ) which auto-phosphorylates and then acts as a tyrosine kinase to phosphorylate members of a family of molecules collectively called Insulin Receptor Substrates (IRSs) (White and Kahn 2021; Saltiel 2021). Nine IRS molecules have been identified. Once phosphorylated, each IRS contributes to downstream activation of a number of different pathways, most of these pathways also respond to signals initiated by other mediators that are independent of both the IR and the different IRS molecules (Saltiel 2021). However, the IR responds only to insulin and, to a lesser degree, Insulin-like Growth Factor-1 (IGF-1) (Hakuno and Takahashi 2018). Therefore, insulin-mediated effects result from IRβ-induced tyrosine phosphorylation of an IRS (White and Kahn 2021). Thus, auto-phosphorylation of IRβ and of members of the IRS family most directly reflect the molecular activity of insulin and can serve as molecular markers of insulin activity.

The two best characterized IRSs are IRS-1 and IRS-2. Both are expressed in many tissues. IRS-1 predominates in skeletal muscle and modulates the anabolic effects of insulin; some IRS-2 is also present although its actual contribution is far less important. Similarly, while some IRS-1 is present in the liver, IRS-2 predominates in hepatocytes and pancreatic β cells and is responsible for much of insulin’s gluco-regulatory activity (Eckstein et al. 2017). The above-mentioned metabolic processes are attenuated in sepsis; however, the molecular mechanisms underlying these effects are unknown. We hypothesize that sepsis attenuates insulin-stimulated tyrosine phosphorylation of either IRβ, IRS-1 or IRS-2. In the studies presented here, we tested this postulate using murine cecal ligation and puncture (CLP), which mimics many characteristics of human sepsis (Osuchowski et al. 2018).

## Materials and methods

### Aim, design and setting

This study tested the hypothesis that CLP impairs phosphorylation of IRβ and IRS-2 in skeletal muscle and of IRβ and IRS-2 in liver. The study design was an evaluation of results from laboratory mice conducted. All studies were approved by the Institutional Animal Care and Use Committee (IACUC) at the Feinstein Institute for Medical Research (IACUC protocol #2018-32), where the study was conducted, adhered to National Institutes of Health Guidelines and met Animal Research: Reporting In Vivo Experiments (ARRIVE) criteria.

### Mice

Studies were performed in 12 week-old male C57BL/6 mice weighing between 25 and 29 g (Jackson Labs, Farmington, CT). Mice were acclimated and maintained in a conventional, light-cycled facility for at least 1 week prior to use. Animals had ad libitum access to food and water except for 6 h. prior to CLP and for 6 h. prior to obtaining blood samples (with or without insulin administration) for fasting glucose levels.

### Cecal Ligation and Puncture (CLP)

CLP with two 22-gauge punctures was performed under isoflurane anesthesia as previously described (Abraham et al. 2018). Animals were resuscitated with 50 mL/kg sterile saline injected subcutaneously after surgery. Fluid administration was repeated at 23 h and, where appropriate, 47 h post-CLP. Imipenem/cilastatin (0.5 mg/kg) was administered subcutaneously at the same time as fluids. Mice were euthanized by cervical dislocation at T_0_ and at 24- and 48-h post CLP. Blood was obtained via cardiac puncture immediately post-mortem and spun to isolate serum. Organs were harvested, homogenized in buffer containing protease and phosphatase inhibitors, aliquoted and stored at − 80 °C (Abcejo et al. 2011).

### Stimulation of insulin signaling

To stimulate Irβ/IRS phosphorylation, a unique cohort of mice received an intraperitoneal (IP) injection of 5U of insulin (Humalog, Lilly USA, Indianapolis, IN USA) 1 h prior to CLP (T_0_ -1) and at 23, and 47 h. post-CLP. One h after the injection the animals were euthanized. Blood and tissue were obtained, homogenized, aliquoted and frozen as above.

### Extraction of protein and determination of concentration

Total tissue lysates were prepared in RIPA buffer (89901, Thermo Scientific, Waltham, MA) containing protease (A32955, Thermo Scientific, Waltham, MA) and phosphatase (A32957, Thermo Scientific, Waltham, MA) inhibitors. Tissue was homogenized using handheld homogenizer (Omni International TH, Kenasaw, GA) and centrifuged at max speed (12,700 rpm) for 20 min. Aliquots were prepared and stored − 80 °C. The total protein concentration in aliquots of skeletal muscle and liver homogenate was determined using the bicinchoninic acid protein assay (23225, Thermo Fisher Scientific, Waltham, MA) as per manufacturer’s instructions.

### Determination of IRβ, IRS-1 and IRS-2 concentrations in tissue

Commercially available ELISA kits were used to determine concentrations of Irβ, IRS-1 and IRS-2 in samples containing 40 µg of protein isolated from either skeletal muscle or liver homogenate (MyBioSource Life Science Resources, San Diego, CA) per the manufacturer’s instructions. Spectrophotometric absorbance (450 nm) was measured in triplicate for all samples.

### Determination of phospho-IRβ (pIRβ), phospho-IRS-2 (pIRS-1) and pIRS-2 levels in tissue

The relative abundance of pIRβ, pIRS-1 and pIRS-2 in 40 µg of protein isolated from skeletal muscle or liver homogenate was determined using sandwich ELISA (Cell Signaling, Danvers MA) per the manufacturer’s instructions. Spectrophotometric absorbance was read at 450 nm.

### Immunoblotting of tissue lysates

Polyacrylamide gel electrophoresis was performed as previously described (Abraham et al. 2018) using 60 μg of protein lysate/lane and precast gels (4–15% gradient) (Bio-Rad, Hercules, CA). IRS-1 was identified with a primary rabbit polyclonal antibody to mouse IRS-1 (2382S; Cell Signaling Technology, Danvers, MA). IRS-2 was identified with a primary rabbit polyclonal antibody to mouse IRS-2 (4502S; Cell Signaling Technology, Danvers, MA). Equal protein loading in each lane was demonstrated using a primary rabbit monoclonal antibody to mouse α-tubulin (2144S; Cell Signaling Technology). Signal intensity was detected using a Goat Anti-Rabbit IgG H&L (HRP) (ab205718, Abcam; Cambridge, UK) visualized with enhanced detection (ChemiDoc MP System and associated Image Laboratory Software 5.2.1; Bio-Rad).

### Measurement of glucose concentration in serum

Serum glucose concentration was determined using a handheld glucometer (LifeScan Europe, Zug, Switzerland).

### Measurement of insulin concentration in serum

Insulin levels were determined by ELISA (Thermo-Fisher, Waltham, MA, USA) per the manufacturer’s instructions.

### Statistics

Results were analyzed using one way or two-way ANOVA with Tukey’s post-hoc correction. The level of significance was set at P < 0.05.

## Results

### CLP Does not affect the concentration of IRβ, IRS-1 or IRS-2 in skeletal muscle or liver tissue

**Fig. 1:**
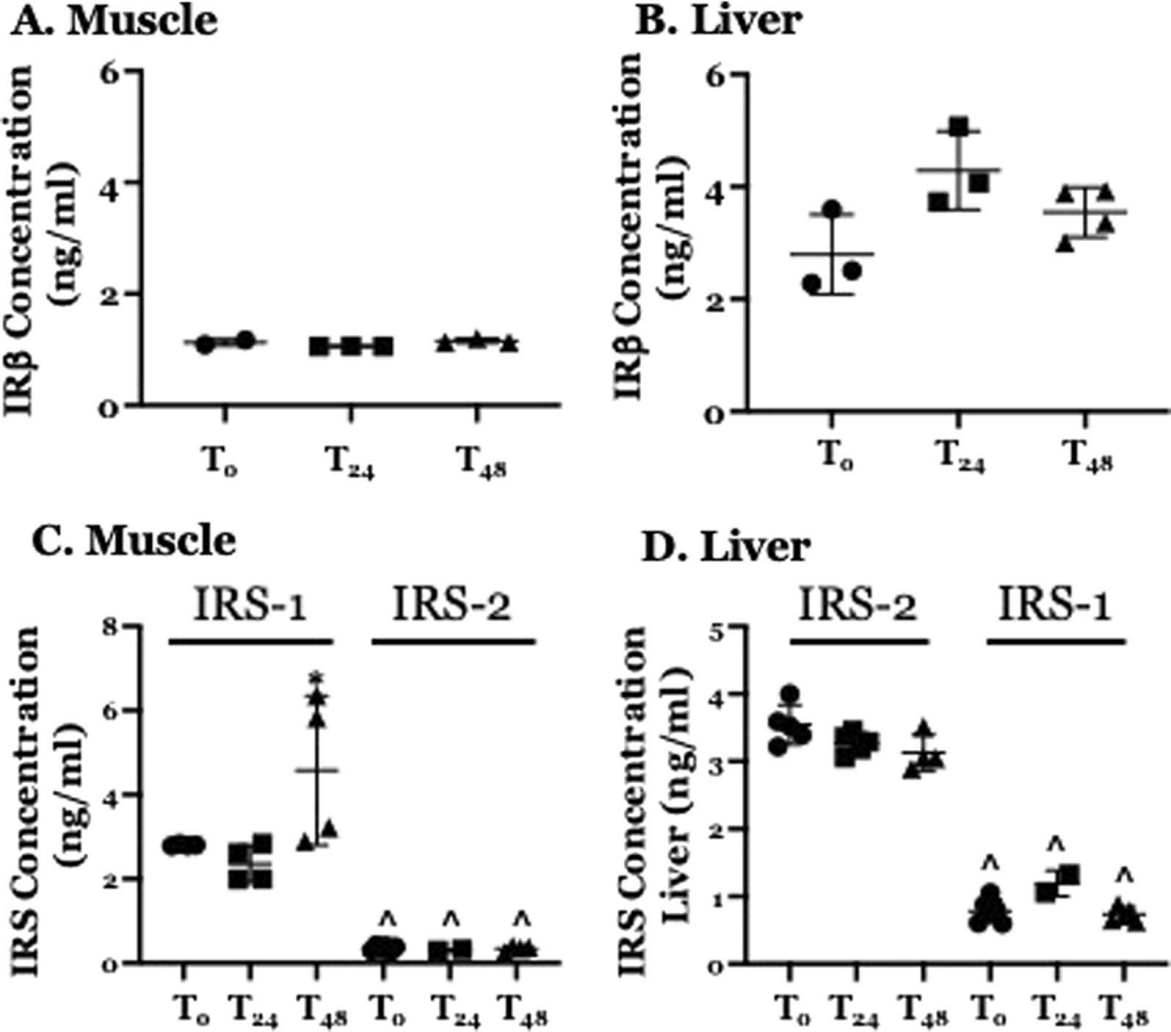
Effect of CLP on the abundance of IR (Insulin Receptor) β and IRS (IR Substrate)-1 in Skeletal Muscle and IRβ and IRS-2 in liver. T: time; subscript indicates time (hrs) post-CLP. Each point represents a measurement in a single animal, heavy bars − mean, light bars − ± standard deviation. Measurements performed using ELISA. Significance determined using one-way ANOVA with Tukey’s post-hoc correction. *Statistically significant relative to concentration at T_0_, **A** Effect of CLP on total IRβ abundance in skeletal muscle. N = 2–3 at each time point. **B** Effect of CLP on total IRβ abundance in Liver. N = 3–4 at each time point. **C** Effect of CLP on total IRS-1/IRS-2 abundance in Skeletal Muscle. N = 2–4 at each time point. ^Statistically significant relative to concentration of IRS-1 at the same time point. **D** Effect of CLP on total IRS-2/IRS-1 abundance in Liver. N = 4–5 at each time point. ^Statistically significant relative to concentration of IRS-2 at the same time point

Levels of IRβ, IRS-1 and IRS-2 in skeletal muscle and liver were measured at baseline and at 24 and 48 h. post-CLP. CLP did not significantly affect IRβ concentration in either skeletal muscle (Fig. 1A) or liver (Fig. 1B). At 48 h. post-CLP, the concentration of IRS-1 in skeletal muscle was significantly higher than that observed at either T_0_ or at 24 h. post-CLP (Fig. 1C). The concentration of IRS-1 in liver and of IRS-2 in both skeletal muscle and liver and at either post-CLP time point was indistinguishable from the concentration at T_0_ (Fig. 1C, D). The concentration of IRS-2 in skeletal muscle was between 5- and 13-fold lower than the concentration of IRS-1 at the same time point, a difference that was highly significant (Fig. 1C). Similarly, the concentration of IRS-1 in liver was between 3- and 4.5-fold lower than that or IRS-2 at the same time point (Fig. 1D). We concluded that 1) decreased insulin signaling could not result from a CLP-mediated differences in the concentration of IRβ, IRS-1 or IRS-2 in either skeletal muscle or liver and 2) the low concentrations of IRS-2 in skeletal muscle and of IRS-1 in liver suggest that these proteins do not contribute to CLP-induced differences in the molecular response to insulin.

### CLP increases insulin-mediated phosphorylation of IRβ in skeletal muscle and liver

**Fig. 2:**
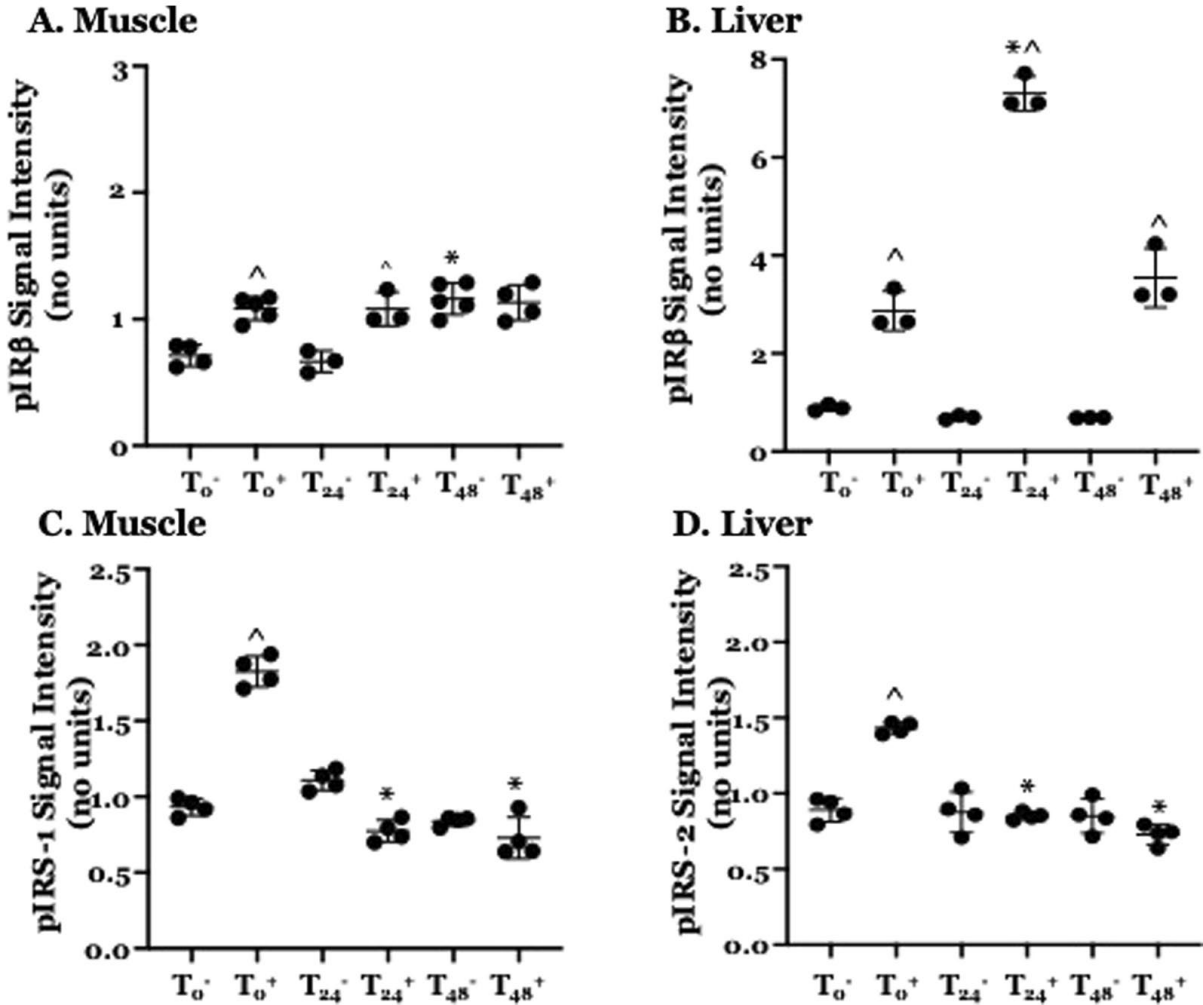
Effect of CLP ± exogenous insulin on the abundance of Phospho (p)IRβ and pIRS-1 in Skeletal Muscle and pIRβ and pIRS-2 in Liver. T_0_: unoperated control; subscript indicated time (hrs) post-CLP. Superscript; –/+ indicates without or with insulin. Each point represents a measurement in a single animal, heavy bars − mean, light bars − ± standard deviation. Measurements performed using ELISA. Value at T_0_ arbitrarily set at 1, other values normalized to T_0_ value. Significance determined using two-way ANOVA with Tukey’s post-hoc test for multiple comparisons. *Statistically significant relative to intensity of animals receiving the same treatment (with or without insulin) at T0, ^Statistically significant relative to intensity at the same time point without insulin. For clarity, other significant differences (eg, relative to T_24_ w/o insulin) not indicated. **A** Effect of CLP on tyrosine phosphorylation of IRβ in skeletal muscle without or in the presence of exogenous insulin. N = 3–5 for each group. **B** Effect of CLP on tyrosine phosphorylation of IRβ in liver without or in the presence of exogenous insulin. N = 3 for each group. **C** Effect of CLP on tyrosine phosphorylation of IRS-1 in skeletal muscle without or in the presence of exogenous insulin. N = 4 for each group. **D** Effect of CLP on tyrosine phosphorylation of IRS-2 in liver without or in the presence of exogenous insulin. N = 4 for each group

The first reaction in the intracellular insulin signaling pathway is tyrosine autophosphorylation of the β subunit of the insulin receptor. Therefore, we examined the effects of CLP on insulin-induced tyrosine phosphorylation of pIRβ in skeletal muscle and liver homogenate. pIRβ abundance in skeletal muscle homogenate was significantly higher following insulin administration than the abundance in untreated animals at T_0_ and at 24 h. post-CLP (Fig. 2A). Insulin administration did not affect IRβ phosphorylation at 48 h. post-CLP. However, the pre-insulin abundance of pIRβ in skeletal muscle homogenate at this time point was higher than that observed at both T_0_ and at 24 h. post-CLP (Fig. 2A). CLP alone did not affect pIRβ abundance in liver homogenate (Fig. 2B). However, hepatic pIRβ abundance was higher in mice receiving insulin than in untreated animals at all three time points studied (Fig. 2B).

### Insulin-mediated phosphorylation of IRS-1 in skeletal muscle and of IRS-2 in liver are decreased following CLP

The key step in Insulin-mediated signal transduction is phosphorylation of IRS-1 in skeletal muscle and of IRS-2 in the liver. Therefore, we examined the effects of CLP on the abundance of pIRS-1 in skeletal muscle homogenate and of pIRS-2 in liver homogenate. In the absence of exogenous insulin, levels of pIRS-1 in muscle (Fig. 2C) and of pIRS-2 in liver (Fig. 2D) at T_0_ and at 24 and 48 h. post-CLP were statistically indistinguishable from each other. One hr. after insulin administration to T_0_ controls, the abundance of pIRS-1 in muscle homogenate and of pIRS-2 in liver homogenate was significantly higher than that observed in untreated post-CLP animals. In contrast, one hr. after insulin treatment, skeletal muscle pIRS-1 abundance in mice that were either 24 or 48 h. post-CLP could not be statistically distinguished from levels in untreated animals (Fig. 2C). Similarly, at both 24 and 48 h. post-CLP, pIRS-2 abundance in liver homogenate obtained 1 h. following insulin administration was not statistically different from the abundance measured in untreated animals at the same post-CLP time points (Fig. 2D). Importantly, serum insulin levels 1 h. following IP insulin administration were significantly higher than levels in untreated controls (data not shown), indicating that IP insulin was absorbed. These findings demonstrate that administration of insulin increased IRS-1 and IRS-2 phosphorylation in T_0_ controls but that CLP attenuated insulin-stimulated phosphorylation of these two proteins.

### Serum glucose levels are lower and serum insulin levels are higher following CLP

**Fig. 3:**
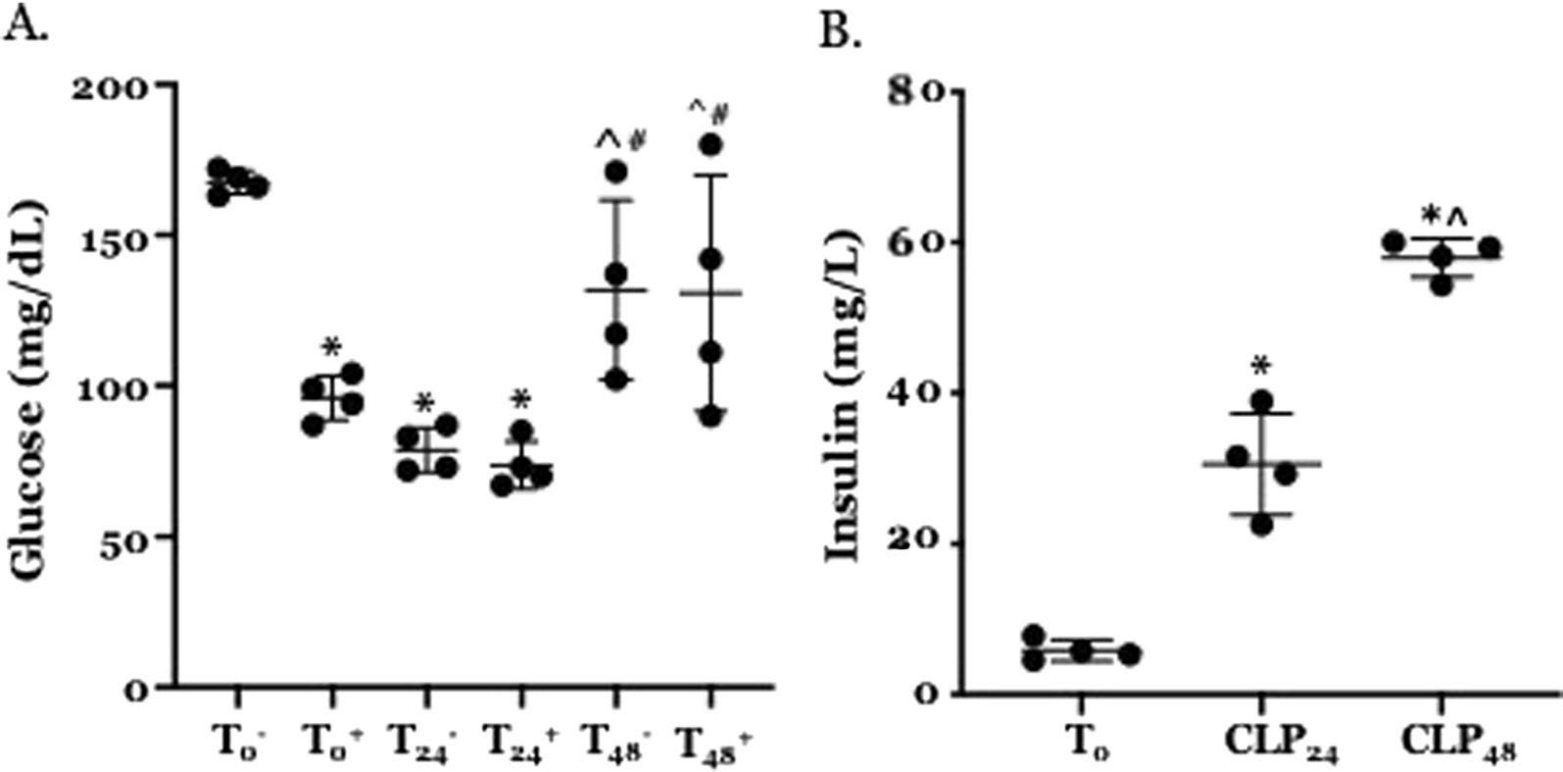
Effects of CLP on serum glucose and insulin levels. T: unoperated control, subscript indicated time (hrs) post-CLP. Each point represents a measurement in a single animal, heavy bars − mean, light bars − ± standard deviation. N = 4 for each time point/condition. **A** Effect of CLP on serum glucose concentration. Significance determined using two-way ANOVA with Tukey’s post-hoc test for multiple comparisons. *Statistically significant relative to T_0_ w/o insulin, ^statistically significant relative to T_24_ w/o insulin, ^#^statistically significant relative to T_24_+ insulin. **B** Effect of CLP on serum insulin concentration. Statistical significance determined using one-way ANOVA with Tukey post-hoc correction for multiple comparisons. *Statistically significant relative to T0, ^statistically significant relative to T_24_

CLP-induced attenuation of IRS tyrosine phosphorylation might result from either hypoglycemia or low levels of endogenous insulin. Therefore, we examined serum glucose and insulin concentrations at T_0_ and following CLP. Serum glucose levels were significantly lower than T_0_ at 24 h. post -CLP but by 48 h. post-CLP were not distinguishable from levels observed in T_0_ mice (Fig. 3A). At T_0_, glucose levels in mice that received insulin were significantly lower than levels in untreated animals. At both post-CLP time points, glucose levels in mice that received insulin could not be distinguished from levels in untreated animals. Serum insulin levels at 24 and 48 h. post-CLP were significantly higher than levels at T_0_ (Fig. 3B). These findings indicate that the low levels of pIRS-1 and pIRS-2 following CLP did not result from either hypoglycemia or hypo-insulinemia.

## Discussion

This study demonstrates impaired tyrosine phosphorylation of IRS-1 in skeletal muscle and of IRS-2 in liver at 24 and 48 h. following CLP, the most commonly-used animal model of human sepsis. The differences did not result from a decrease in total levels of either protein and were noted despite circulating insulin levels that were higher than those observed at T_0_. Further, in contrast to animals not subjected to CLP, a substantial dose of exogenous insulin did not affect levels of either pIRS-1 in skeletal muscle or of pIRS-2 in liver. These findings identify a molecular correlate for CLP-induced insulin resistance that may be present in septic humans.

Sepsis has long been described as a state of hormonal, and in particular insulin, resistance. This designation reflects the observation that the insulin-induced change in serum glucose levels in a septic patient was significantly less than that observed following administration of a similar dose to a non-septic patient (Dahn et al. 1985). However, insulin is not the sole determinant of the serum glucose concentration. Rather, the concentration may also be profoundly affected by the complex endocrine and inflammatory milieu of sepsis. Thus, inflammation/CLP/sepsis-induced increases in endogenous levels of epinephrine, glucagon, cortisol, and pro-inflammatory cytokines and treatments such as norepinephrine administration may limit the ability to assess the effects of insulin. One alternative approach to identifying hormone resistance, employed here, is to examine the ability of exogenously-administered hormone to activate a cellular or molecular pathway that is not directly affected by counter-regulatory mechanisms. A similar approach was used to identify sepsis-induced decreases in the activation of G-protein coupled receptors by catecholamines, growth hormone and glucagon, and in assessing the abundance of the α isoform of the glucocorticoid receptor (Yang et al. 1997; Rehman et al. 2020; Yumet et al. 2006; Hsu et al. 1999; Abraham et al. 2018; Deutschman et al. 1995). Importantly, only insulin and IGF-1 stimulate tyrosine phosphorylation of IRS-1/2 (Hakuno and Takahashi 2018). Pruekprasert et al. reported that CLP reduced IGF-1, which is expressed in the liver in response to GH (Pruekprasert et al. 2021). Of note, our previous work demonstrated reduced GH levels post-CLP (Abraham et al. 2018). Thus, the lower levels of phospo-IRS-1/2 following CLP do not reflect decreased stimulation by IGF-1. Conversely, our demonstration that CLP attenuated IRS-1/2 tyrosine phosphorylation indicates that CLP, and perhaps sepsis, are indeed states of insulin resistance.

Attenuated IRS phosphorylation is consistent with some of our previously reported findings following CLP. We have demonstrated that CLP reduced phosphorylation of gp130, the protein that initiates the intracellular response to IL-6 (Abcejo et al. 2009). Further, we found that phosphorylation of the retinoblastoma protein, which is essential for cellular regeneration in hepatocytes, was lower than baseline following CLP (Abcejo et al. 2011). Others have identified reduced phosphorylation of cardiac and skeletal muscle proteins, and inactivation of protein kinase C in liver (Wu et al. 2001; Shimada et al. 2022; Lang and Frost 2007). Conversely, some studies suggest that phosphorylation in inflammatory pathways contributes to the pathogenesis of sepsis (Gao et al. 2022; Lv et al. 2021). Further exploration is warranted.

Additional explanations for impaired IRS phosphorylation must be considered. The data presented here (Figs. 1 and 2) indicate that reduced phosphorylation cannot be explained by a decrease in the overall concentrations of IRS-1 in muscle or of IRS-2 in liver. Hypoglycemia itself can limit IRS phosphorylation but this effect is mediated by hypo-insulinemia and thus is not consistent with the findings presented in Fig. 3 or with those reported by others (Ferreira et al. 2017). As mentioned, IGF-1 can induce IRS phosphorylation but intrahepatic IGF-1 abundance is reduced post CLP, as are blood levels of GH, which stimulates IGF-1 expression (Hakuno and Takahashi 2018). Further, resistance to GH/IGF-1 – mediated IRS phosphorylation at 24 h. post CLP is consistent with the peripheral resistance to pituitary hormones that characterizes the early phase of critical illness, including sepsis (Téblick et al. 2022).

Attenuated IRS phosphorylation following CLP might reflect the anti-phosphorylation activity of several intracellular proteins activated by inflammation (Emanuelli et al. 2001; Rui et al. 2002; Ueki et al. 2004; Wada et al. 2011). Alternatively, we and other have identified a sepsis/CLP-induced reduction in oxidative phosphorylation (Levy and Deutschman 2007; Brealey et al. 2002). While this work indicates that auto-phosphorylation is not affected by CLP, transfer of a phosphate group from the receptor to the IRS could be attenuated. This mechanism would be consistent with our demonstration of reduced transfer of a phosphate from gp130, the IL-6 receptor, to SATA-3, th intracellular mediator of IL-6 activity (Abcejo et al. 2009). These transfers are energy requiring reactions, which are affected by sepsis/CLP. Finally, lower levels of pIRS-1/2 could result from enhanced phosphatase activity. However, several studies indicate that, in general, activity of these enzymes is reduced post-CLP and during clinical sepsis (Jacob et al. 2010; Zhao and Huang 2019; Clavier et al. 2019; Heun et al. 2019).

Liver and skeletal muscle each express both IRS-1 and IRS-2. However, it is well-documented that IRS-1 is the major effector of insulin activity in skeletal muscle while IRS-2 serves the same function in liver (White and Kahn 2021; Saltiel 2021). The data presented here are consistent with those conclusions; the amount of IRS-2 in skeletal muscle and of IRS-1 in liver is far lower than that of IRS-1 and IRS-2, respectively. Thus, it is unlikely that either is a substantial contributor to CLP- or sepsis-induced attenuation of insulin activity. Indeed, this conclusion led to our decision not to examine the effect of CLP on phosphorylation of mISR-2 in skeletal muscle or of IRS-1 in liver.

Additional comments on the data presented here are necessary. The most important is that sepsis is a distinctly human disorder that, to date, cannot be adequately reproduced in animals. While CLP is the “best” and most commonly used model, it lacks many features of the clinical disorder (Osuchowski et al. 2018). For example, hyperglycemia is the norm in human sepsis while mice become relatively hypoglycemic, as demonstrated here. In addition, therapeutic approaches routinely employed in treating human sepsis-supplemental oxygen, mechanical ventilation and surgical excision of the source of infection—are rarely used following CLP. All of our measurements were made on CLP survivors, which biases results. We used T_0_ (non-septic) mice as controls. At one time sham operation on a separate cohort of animals was routinely included in CLP studies. However, we have eliminated this approach at the behest of our IACUC because, within 6 h. post-op, findings in sham operated animals no longer differ from those in T_0_ controls. The earliest CLP-induced abnormalities we have noted have been at 16 h. post procedure, when the effects of sham operation are no longer detectable. Finally, recent work indicates that the limited memory T cell diversity present in laboratory mice affects the response to CLP and other infectious/inflammatory events (Taylor et al. 2020; Hamilton et al. 2020; Beura et al. 2016). In contrast, the memory T cell compartment in septic patients is robust. Thus, extrapolating findings derived from murine CLP to human sepsis must be done with caution, especially given that the ability to directly examine liver or muscle tissue in patients is quite limited.

That said, demonstration of impaired IRS phosphorylation post-CLP may have clinical ramifications. The findings presented here provide yet another example of sepsis-associated endocrine resistance (Téblick et al. 2022). It has been suggested that the large doses of bioactive amines frequently used to correct sepsis-induced hypotension may be detrimental (Andreis and Singer 2016). The same may be true of using large doses of insulin to correct hyperglycemia in sepsis. Both norepinephrine and insulin can be anti-inflammatory (Niekerk et al. 2020; Stolk et al. 2020). Thus, use of either drug, especially in pharmacologic doses, in a state where profound immunosuppression has been pathobiologically implicated may be problematic (Torres et al. 2022). Studies by van den Berghe’s group have demonstrated that the endocrinopathy of critical illness is quite complex and its treatment may require intervention beyond simply providing exogenous hormones (Téblick et al. 2022).

## Conclusion

In conclusion, our findings indicate that insulin resistance, like other endocrine abnormalities that accompany sepsis, reflects a defect in a transcellular signaling system. This pathway mediates multiple properties, is present in a variety of cells, and is operative in several organ systems. Thus, titrating insulin to normalize one clinical manifestation of sepsis-hyperglycemia—may over-emphasize correction of this specific insulin-mediated effect and neglect others of greater pathobiological importance. A concern for unintended consequences suggests that exogenous insulin be used cautiously.

## Data Availability

The datasets used/analyzed during the current study are available from the corresponding author on reasonable request.

## Abbreviations

IRβ: β-Subunit of the insulin receptor
IRS: Insulin receptor substrate
CLP: Cecal ligation and puncture
pIRβ and pIRS: Phospho-IRb and phospho-IRS
IGF-1: Insulin-like Growth Factor
IP: Intraperitoneal
